# Supplementary material for: Effect of the Surface Hydrophobicity Degree on the In Vitro Release of Polar and Non-Polar Drugs from Polyelectrolyte Matrix Tablets
Source: Polymers (Basel). 2018 Nov 27;10(12):1313. doi: 10.3390/polym10121313 (PMC6401953; doi:10.3390/polym10121313)
Supplement: Supplementary file 1 [file polymers-10-01313-s001.pdf]

Supplementary

1. FTIR spectra of PAM-0, PAM-0Na; PAM-18 and PAM-18Na

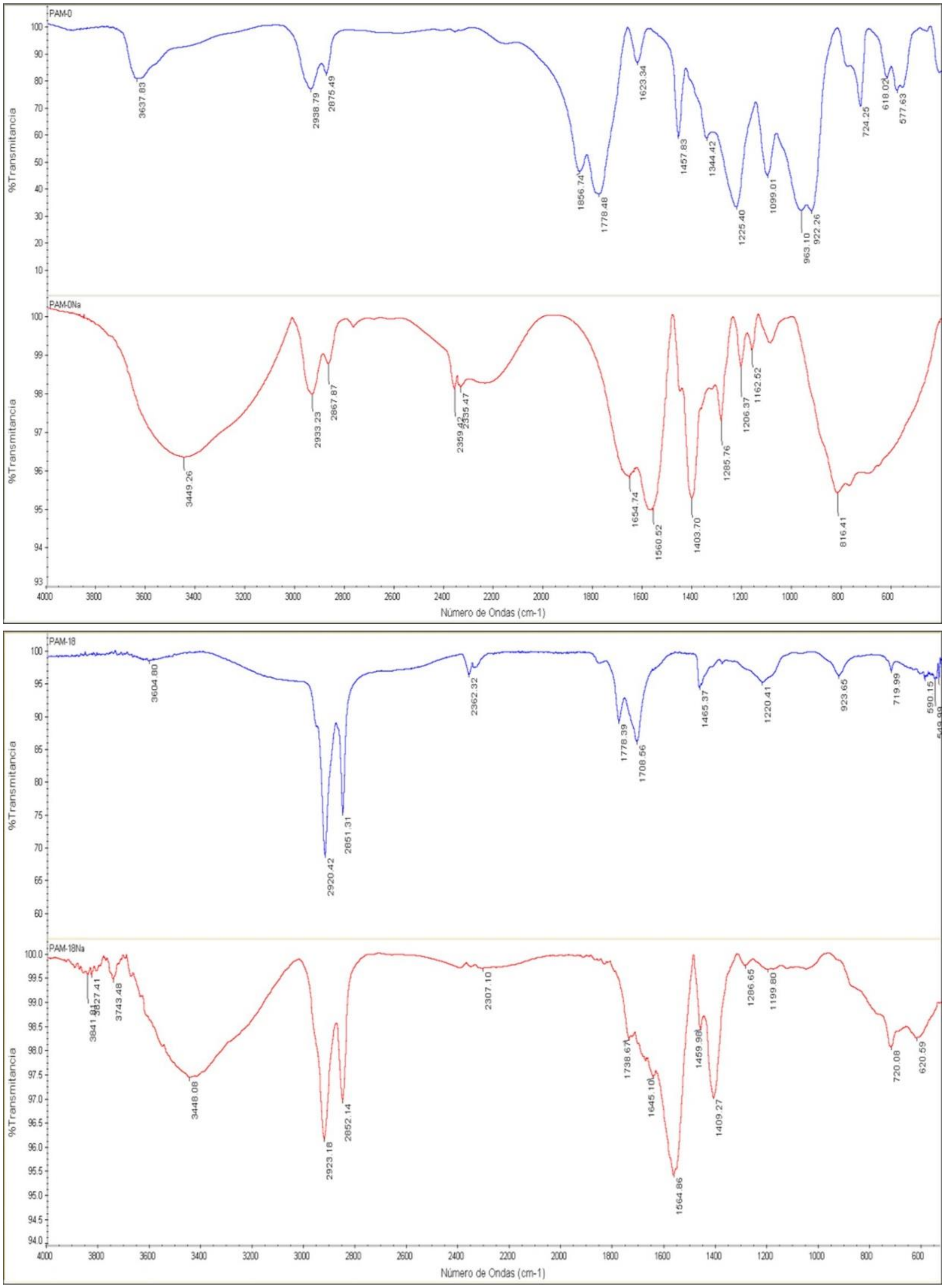

Figure 1. *FTIR spectra of PAM-0, PAM-0Na; PAM-18 and PAM-18Na*

2. Analysis of the roughness of the surface of the tablets

Determining the roughness degree for each tablet was carried out by the micro-display high magnification technique using a micro-stereoscope (Nikon SMZ1500, Nikon Industries Inc., Melville, NY, USA). The "surface roughness" was estimated with the NIS-Elements Advanced Research software (Nikon Industries Inc., Melville, NY, USA). For this, several images of each tablet were captured and used to analyze the contrast of pixels in light and dark areas under the following conditions: region of interest (ROI) 189×120 pixels, binary threshold, function intensity (left = 90), (right 200) and 0.75x optic zoom. All tests were performed under homogeneous conditions of incident light intensity, temperature and relative humidity. Finally, the relative roughness index (IR/A) indicates the surface roughness of the tablets and it is defined as:

$$I_{R/A} = \frac{\left(\frac{ANR}{R}\right)}{ANR} = \frac{1}{R} \qquad (1)$$

Where ANR is the not roughened area of the image and R is the roughness factor, both parameters given by the software. When $I_{R/A} \leq 1.20$, it is established that the surface tends to be rough, while $I_{R/A} \geq 1.30$ suggests that the surface is smooth. Furthermore, values between 1.20 and 1.30 set an intermediate state between smooth and rough surface.

Results:

The study carried out on the surfaces of the tablets by the micro-visualization technique showed that the degree of roughness depends both on the type and amount of polymer used and on the drug under study. **Figure 1** shows the microphotographs and software image analysis for the surfaces of the tablets of Carbamazepine and metoprolol, with the materials PAM-18Na, PAM-0Na, and HPMC at different proportions. In the upper left part of each image, the value of the roughness index ($I_{R/A}$) is shown.

In the case of the tablets of carbamazepine without polymer, the value of $I_{R/A}$ was 1.21 indicating the formation of a rough surface. In the case of the carbamazepine was mixed with PAM-0Na, PAM-18Na and HPMC, the values of $I_{R/A}$ were between 1.08 and 1.22 suggesting that the tablet surfaces tend to become slightly rougher regardless of the type and amount of polymer used. On the other hand, the metoprolol succinate tablets without polymer. In the case of metoprolol tablets, it is observed that the $I_{R/A}$ = 1.39 and with that, that its surface is smooth. When you have the mixture with the other polymeric materials, it is observed that increasing the polymer ratio decreases the $I_{R/A}$, indicating that the tablets tend to have greater roughness on its surface, being the most marked effect for the reference material HPMC.

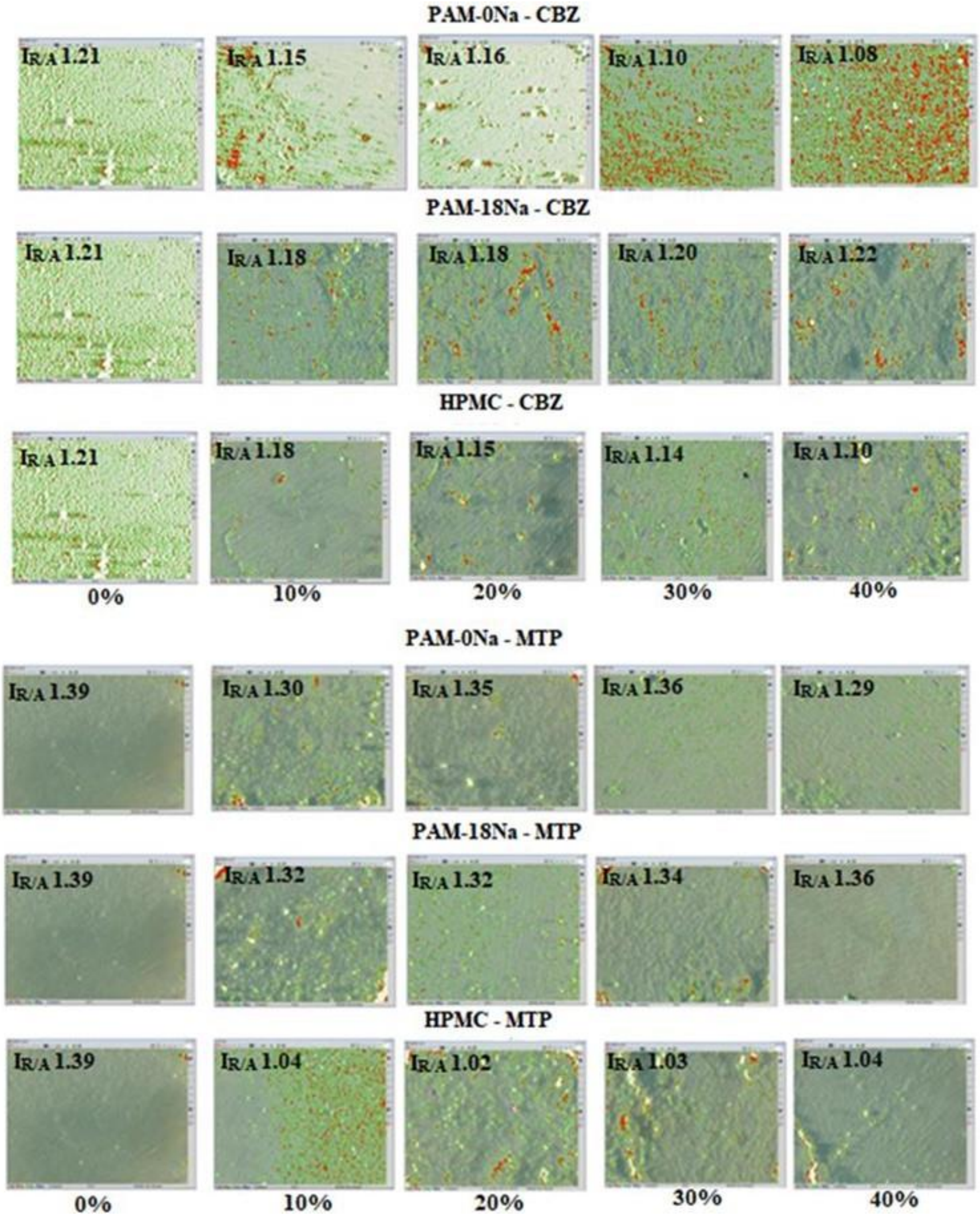

Figure 2. Surface of tablets of Carbamazepine (CBZ) and metoprolol (MTP) to different proportions of polymers (The combination of green and red indicates rough surface, while the single green color indicates smooth surface).

3. Data of the thermodynamics surface analyzes by contact angle and WORK model

Table1. Contact angles for water, ethyleneglycol and Isopropanol

System	Drug	Polymer (%)	Contact angle (°)		
			**Water**	**Ethyleneglycol**	**Isopropanol**
**PAM-0Na**	**CBZ**	0	100.5 ± 0.25	73.4 ± 4.2	20.4 ± 1.0
		10	91.1 ± 2.3	83.9 ± 3.1	22.3 ± 5.6
		20	87.9 ± 0.5	72.0 ± 6.4	18.1 ± 0.1
		30	76.6 ± 2.1	65.3 ±5.7	14.4 ± 0.1
		40	62.2 ± 0.5	47.0 ± 5.0	17.3 ± 0.1
**PAM-18Na**		0	100.5 ± 0.25	73.4 ± 4.2	20.4 ± 1.0
		10	82.3 ± 2.5	68.4 ± 4.9	19.9 ± 3.0
		20	85.9 ± 2.0	71.5 ± 2.7	19.5 ± 2.1
		30	96.7 ± 1.8	63.5 ± 4.5	15.2 ± 2.1
		40	99.2 ± 5.5	72.8 ± 4.9	15.7 ± 2.9
**HPMC**		0	100.5 ± 0.25	73.4 ± 4.2	20.4 ± 1.0
		10	81.3 ± 3.1	70.3 ± 2.8	11.3 ± 2.4
		20	85.7 ± 1.4	71.2 ± 3.9	16.3 ± 1.2
		30	90.3 ± 1.0	78.2 ± 4.7	13.5 ± 1.2
		40	93.4 ± 2.3	79.9 ± 8.0	18.3 ± 2.4
**PAM-0Na**	**MTP**	0	56.5 ± 6.8	65.3 ± 4.9	21.5 ± 2.2
		10	76.2 ± 2	61.8 ± 1.6	19.2 ± 0.8
		20	45 ± 0.2	63.4 ± 0.3	19 ± 0.1
		30	56.3 ± 2.3	63.6 ± 2.6	22 ± 0.1
		40	81.7 ± 0.8	76.4 ± 0.7	16.8 ± 0.1
**PAM-18Na**		0	56.5 ± 6.8	65.3 ± 4.9	21.5 ± 2.2
		10	63 ± 2.7	73.3 ± 4.7	16.2 ± 1.3
		20	71.1 ± 3	71.6 ± 0.6	18 ± 1.9
		30	78.4 ± 3.7	71.4 ± 4.2	16.2 ± 2.4
		40	84.3 ± 6.6	76.3 ± 5.4	21.3 ± 3.1
**HPMC**		0	56.5 ± 6.8	65.3 ± 4.9	21.5 ± 2.2
		10	74.7 ± 3	71 ± 2.6	22.7 ± 0.4
		20	78.9 ± 2.7	51.5 ± 2.4	19.5 ± 2.2
		30	81 ± 4.2	56.5 ± 2.4	18.3 ± 0.1
		40	89.2 ± 1.3	64.9 ± 4.8	16 ± 0.8

Table 2. Surface free energy (SFE) calculations

Polymer	Drug	Polymer (%)	W_{adh} (mJ/m²)	Surface Free energy SFE (mJ/m²)			(R^2)	(s)	$I_{p/d}$
				SFE_{total}	SFE^d	SFE^p			
PAM-0Na	CBZ	0	58.8 ± 0.3	22.6 ± 1.2	21.0 ± 1.3	1.6 ± 0.1	0.985	1.2	0.1
		10	70.5 ± 2.8	18.9 ± 0.9	12.0 ± 2.0	7.0 ± 1.6	0.903	1.1	0.6
		20	74.6 ± 0.6	22.0 ± 1.3	14.1 ± 1.7	8.0 ± 0.5	0.982	1.7	0.6
		30	88.6 ± 2.6	27.3 ± 1.8	10.3 ± 0.4	16.9 ± 1.6	0.992	1.2	1.6
		40	105.5 ± 0.5	39.4 ± 0.6	7.9 ± 0.7	31.5 ± 0.2	0.998	0.7	4.0
PAM-18Na		0	58.8 ± 0.3	22.6 ± 1.2	21.0 ± 1.3	1.6 ± 0.1	0.985	1.2	0.1
		10	81.6 ± 3.0	24.2 ± 0.2	11.9 ± 2.2	12.3 ± 2.4	0.990	1.5	1.0
		20	77.1 ± 2.5	22.5 ± 1.0	13.0 ± 0.6	9.5 ± 1.4	0.991	0.5	0.7
		30	63.5 ± 2.2	25.0 ± 1.1	22.6 ± 1.5	2.5 ± 0.7	0.951	1.3	0.1
		40	60.4 ± 6.8	23.1 ± 0.3	21.0 ± 1.7	2.2 ± 1.5	0.996	0.6	0.1
HPMC		0%	58.8 ± 0.3	22.6 ± 1.2	21.0 ± 1.3	1.6 ± 0.1	0.985	1.2	0.1
		10%	82.7± 3.9	24.5 ± 1.1	11.7 ± 2.0	12.8 ± 2.8	0.985	1.2	1.1
		20%	77.2 ± 1.8	22.7 ± 0.6	13.4 ± 1.8	9.4 ± 1.4	0.988	1.4	0.7
		30%	71.5± 1.3	20.6 ± 1.0	14.0 ± 1.4	6.6 ± 0.7	0.944	1.6	0.5
		40%	67.6± 2.9	20.0 ± 1.8	14.9 ± 2.9	5.1 ± 1.4	0.912	3.0	0.3
PAM-0Na	MTP	0%	111.4 ± 6.9	45.9 ± 8.4	3.5 ± 2.0	42.4 ± 10.4	0.981	5.1	12.3
		10%	89.0 ± 2.4	25.7 ± 0.7	10.9 ± 0.3	14.8 ± 0.4	0.999	0.0	1.4
		20%	122.8 ± 0.2	59.1 ± 0.2	1.5 ± 0.1	57.6 ± 0.2	0.979	0.1	38.2
		30%	111.7 ± 2.4	45.6 ± 2.5	3.3 ± 0.1	42.2 ± 2.6	0.985	0.4	12.6
		40%	82.3 ± 0.9	23.0 ± 0.3	10.1 ± 0.3	12.8 ± 0.5	0.960	0.3	1.3
PAM-18Na		0%	111.4 ± 6.9	45.9 ± 8.4	3.5 ± 2.0	42.4 ± 10.4	0.981	5.1	12.3
		10%	104.6 ± 3.1	38.1 ± 2.9	3.9 ± 0.1	34.2 ± 2.9	0.958	1.1	8.8
		20%	95.1 ± 3.5	30.6 ± 2.5	6.6 ± 1.0	24.0 ± 3.5	0.973	0.3	3.6
		30%	86.4 ± 4.5	25.6 ± 2.4	9.7 ± 0.6	16.0 ± 3.1	0.978	0.8	1.7
		40%	79.1 ± 8.3	22.5 ± 2.8	10.8 ± 2.3	11.8 ± 4.9	0.963	2.5	1.1
HPMC		0%	111.4 ± 6.9	45.9 ± 8.4	3.5 ± 2.0	42.4 ± 10.4	0.981	5.1	12.3
		10%	90.9 ± 3.6	28.0 ± 2.3	7.6 ± 0.6	20.4 ± 2.8	0.981	0.4	2.7
		20%	85.7 ± 3.3	28.1 ± 1.1	14.4 ± 1.5	13.7 ± 2.4	0.982	1.0	0.9
		30%	83.2 ± 5.2	26.8 ± 1.5	14.5 ± 1.8	12.3 ± 3.3	0.991	0.4	0.8
		40%	72.9 ± 1.6	23.6 ± 1.1	17.1 ± 0.6	6.5 ± 0.5	0.993	0.8	0.4

4. Drug Release Kinetic Models

Data obtained from the in vitro dissolution study were analysed using the zero order, first order, Higuchi[1–3] and Korsmeyer-Peppas models [4–6]. The Higuchi is widely used to describe the release of soluble and sparingly soluble drugs in aqueous media, from various solid matrices according to the equation:

$$Q_t = k_H t^{1/2} \quad (2)$$

where k_H is the Higuchi dissolution constant, while Qt correspond to the concentration released at time t.

The Korsmeyer–Peppas model is a generalized model that allows to explain drug delivery mechanisms where erosion and/or dissolution of the polymeric matrix occurs. The related equation is:

$$\frac{M_t}{M_\infty} = k_r t^n \qquad (3)$$

where Mt/M∞ corresponds to the fraction of drug released at time t; kr is the release constant representative of polymer-drug interactions, n is the diffusion exponent that is characteristic for the release mechanism. When n equals 0.5, the equation becomes equal to the Higuchi model, indicating that the release mechanism is of a Fickian type (case I), while values of n between 0.5 and 1.0 suggest that the release mechanism corresponds to an anomalous (non-Fickian) transport. Values of 1.0 indicate that the release mechanism is similar to a zero order release, while values of n greater than 1.0 (Super Case II transport), suggest a drug release process dependent of the relaxation of the polymer chains in the matrix, passing from a vitreous state (lower kinetic movement and increased potential energy) to a relaxed state rubber type (high kinetic movement and lower potential energy).

Results:

The results of the release kinetic models of CBZ and MTP from tablets elaborated with PAM-0Na, PAM-18Na and HPMC polymers at different polymer proportions are summarised in **Table 3**. According to the kinetic study of the release profiles of CBZ and MTP, for CBZ with PAM-0Na in both dissolution media, the data fit the Higuchi model at polymer proportions of 30% and 40%, suggesting that the release mechanism is apparently controlled by the drug from the compressed matrix towards the bulk and does not depend on the polymer (Fickian diffusion) [7,8]. In the case of CBZ with PAM-18Na, very similar results were observed to those previously obtained with an analogous polymeric material (PAM-18K) [6,9] in gastric media, in which the data fit very well to Higuchi's model, suggesting that the CBZ release is given by the Fickian diffusion process. On the contrary, in duodenal media, the data fit to the Korsmeyer-Peppas model. According to this same model, n = 0.5-1.0 suggests that the mechanism of release is anomalous, controlled by the relaxation of the polymer chains, going through a process where the dissolution media penetrates the compressed matrix forming pores and then erode it. With values of n > 1, the mechanism is of super transport type II, where the polymer matrix makes a transition, from a glassy state of low and very cohesive low kinetic movement to a relaxed rubber type of higher kinetic energy and less cohesive [10–14]. This pH-dependent behaviour suggests that the ionic characteristics of the polymer along with the chain length are the main condition that leads to a specific mechanism of drug release. In the case of CBZ and HPMC, which corresponds to a model material for controlled release, the data obtained using gastric medium with percentages between 10% and 20% did not fit any of the models used. Whilst in the duodenal media with polymer percentages between 20% and 40%, the data are better fitted to a model of first order, which is typical for apolar drug releases, from porous matrices such as those in the study.

In the case of MTP releases, the dissolution profile data did not fit any of the kinetic models evaluated. This result is expected because of the matrix tablets were very porous, soft and erodible, as evidenced in the results of hardness and time of disintegration obtained. In this way, it is necessary to evaluate other types of kinetic models, such as Hopfenberg [15–18] and Hixson-Crowell [19–22] which are more used for this type of matrices.

Table 3A. Kinetic models of drug release for CBZ tablets elaborated with PAM-0Na, PAM-18Na and HPMC polymers at different polymer proportions.

Drug	Polymer	Polymer amount (%)	Media	Zero order		First order		Higuchi		Korsmeyer-peppas		
				k_0	R^2	k_1	R^2	k_H	R^2	n	k_r	R^2
(CBZ)	PAM-0Na	0	Gastric	0.029	0.985	1.00E-03	0.986	0.663	0.927	0.709	6.940	0.798
		10		0.136	0.900	1.00E-03	0.970	3.362	0.987	0.569	0.422	0.967
		20		0.148	0.932	9.00E-04	0.980	3.586	0.983	0.801	1.659	0.974
		30		0.145	0.934	3.00E-03	0.967	3.538	0.980	0.621	0.583	0.975
		40		0.157	0.803	2.00E-03	0.899	4.250	0.917	0.673	0.592	0.922
		0	Duodenal	0.033	0.985	9.00E-04	0.988	0.774	0.961	0.624	3.247	0.869
		10		0.044	0.988	5.00E-04	0.990	1.110	0.969	0.673	2.997	0.974
		20		0.063	0.976	8.00E-04	0.979	1.588	0.977	0.649	1.662	0.987
		30		0.083	0.903	9.00E-04	0.931	1.895	0.964	0.721	1.871	0.920
		40		0.099	0.910	1.00E-03	0.947	2.592	0.980	0.847	2.997	0.947
	PAM-18Na	0	Gastric	0.029	0.985	1.00E-03	0.986	0.663	0.927	0.709	6.940	0.798
		10		0.051	0.988	6.00E-04	0.994	1.303	0.993	0.675	2.470	0.996
		20		0.026	0.944	3.00E-04	0.950	0.672	0.987	0.659	4.227	0.984
		30		0.029	0.986	3.00E-04	0.990	0.726	0.993	0.522	1.688	0.992
		40		0.014	0.944	2.00E-04	0.947	0.375	0.993	0.603	5.089	0.985
		0	Duodenal	0.033	0.985	9.00E-04	0.988	0.774	0.961	0.624	3.247	0.869
		10		0.075	0.831	1.00E-03	0.902	1.999	0.927	0.285	0.091	0.936
		20		0.046	0.947	7.00E-04	0.965	1.241	0.981	0.209	0.081	0.981
		30		0.061	0.778	1.00E-03	0.871	1.641	0.871	0.193	0.049	0.882
		40		0.057	0.879	8.00E-04	0.912	1.507	0.948	0.286	0.128	0.957
	HPMC	0	Gastric	0.029	0.985	1.00E-03	0.986	0.663	0.927	0.709	6.940	0.798
		10		0.01	0.021	1.00E-03	0.940	0.586	0.096	0.062	0.037	0.078
		20		0.081	0.646	9.00E-04	0.634	2.141	0.817	0.445	0.297	0.913
		30		0.082	0.982	1.00E-03	0.987	1.902	0.952	0.847	4.959	0.985
		40		0.05	0.994	2.00E-03	0.993	1.255	0.967	0.819	6.880	0.977
		0	Duodenal	0.033	0.985	9.00E-04	0.988	0.774	0.961	0.624	3.247	0.869
		10		0.151	0.762	7.00E-03	0.956	4.135	0.893	0.424	0.117	0.915
		20		0.140	0.967	2.00E-03	0.988	3.590	0.993	0.640	0.693	0.997
		30		0.078	0.996	1.00E-03	0.993	1.916	0.967	0.745	2.767	0.994
		40		0.050	0.986	6.00E-04	0.992	1.260	0.989	0.876	8.987	0.976

* The squares highlighted in yellow show the semi-empirical release model with better fit.

Table 3B. Kinetic models of drug release for MTP tablets elaborated with PAM-0Na, PAM-18Na and HPMC polymers at different polymer proportions.

Drug	Polymer	Polymer amount (%)	Media	Zero order		First order		Higuchi		Korsmeyer-peppas		
				k_0	R^2	k_1	R^2	k_H	R^2	n	k_r	R^2
(MTP)	PAM-0Na	0	Gastric	-0.001	0.001	2.00E-04	0.009	0.081	0.018	-0.008	0.011	0.049
		10		0.051	0.164	1.00E-03	0.211	1.609	0.297	0.033	0.016	0.189
		20		0.057	0.214	9.00E-04	0.332	1.760	0.372	0.047	0.017	0.448
		30		0.058	0.085	1.00E-03	0.053	2.057	0.180	0.074	0.017	0.358
		40		0.001	0.000	2.00E-03	0.018	0.131	0.010	0.028	0.014	0.105
		0	Duodenal	0.019	0.502	1.00E-03	0.508	0.476	0.470	0.025	0.014	0.345
		10		-0.013	0.096	1.00E-03	0.086	0.293	0.077	-0.016	0.011	0.067
		20		0.007	0.288	4.00E-03	0.041	0.191	0.317	0.010	0.011	0.334
		30		0.060	0.125	1.00E-03	0.115	0.288	0.168	0.019	0.012	0.219
		40		0.011	0.057	1.00E-03	0.015	0.391	0.108	0.035	0.014	0.239
	PAM-18Na	0	Gastric	-0.001	0.001	2.00E-04	0.009	0.081	0.018	-0.008	0.011	0.049
		10		0.061	0.330	2.00E-03	0.394	1.831	0.459	0.187	0.033	0.607
		20		0.064	0.689	2.00E-03	0.651	1.715	0.783	0.146	0.032	0.800
		30		0.051	0.672	1.00E-03	0.732	1.407	0.803	0.160	0.040	0.891
		40		0.039	0.866	6.00E-04	0.879	1.042	0.945	0.161	0.062	0.969
		0	Duodenal	0.019	0.502	1.00E-03	0.508	0.476	0.470	0.025	0.014	0.345
		10		0.104	0.747	3.00E-03	0.877	2.777	0.833	0.281	0.062	0.854
		20		0.039	0.615	2.00E-03	0.615	0.998	0.577	0.074	0.020	0.523
		30		0.058	0.431	2.00E-03	0.450	1.717	0.580	0.179	0.036	0.750
		40		0.029	0.300	5.00E-04	0.314	0.867	0.418	0.148	0.046	0.544
	HPMC	0	Gastric	-0.001	0.001	2.00E-04	0.009	0.081	0.018	-0.008	0.011	0.049
		10		0.083	0.265	1.00E-03	0.319	2.553	0.446	0.091	0.017	0.548
		20		0.119	0.491	9.00E-04	0.616	3.347	0.698	0.232	0.040	0.757
		30		0.104	0.336	1.00E-02	0.572	3.126	0.543	0.170	0.025	0.574
		40		0.148	0.869	6.00E-03	0.879	3.873	0.933	0.268	0.046	0.937
		0	Duodenal	0.019	0.502	1.00E-03	0.508	0.476	0.470	0.025	0.014	0.345
		10		0.016	0.465	8.00E-03	0.117	0.424	0.498	0.021	0.011	0.447
		20		0.027	0.567	2.00E-02	0.019	0.745	0.669	0.041	0.012	0.751
		30		0.129	0.386	4.00E-02	0.685	2.076	0.541	0.164	0.024	0.704
		40		0.104	0.579	7.00E-03	0.764	2.916	0.713	0.257	0.043	0.775

References:

1. Siepmann, J.; Peppas, N. A. In honor of Takeru Higuchi. *Int. J. Pharm.* **2011**, *418*, 1–2, doi:http://dx.doi.org/10.1016/j.ijpharm.2011.07.030.
2. Siepmann, J.; Peppas, N. A. Higuchi equation: Derivation, applications, use and misuse. *Int. J. Pharm.* **2011**, *418*, 6–12, doi:http://dx.doi.org/10.1016/j.ijpharm.2011.03.051.
3. Brophy, M. R.; Deasy, P. B. Application of the Higuchi model for drug release from dispersed matrices to particles of general shape. *Int. J. Pharm.* **1987**, *37*, 41–47, doi:http://dx.doi.org/10.1016/0378-5173(87)90008-1.
4. Korsmeyer, R. W.; Gurny, R.; Doelker, E.; Buri, P.; Peppas, N. A. Mechanisms of solute release from porous hydrophilic polymers. *Int. J. Pharm.* **1983**, *15*, 25–35, doi:http://dx.doi.org/10.1016/0378-5173(83)90064-9.
5. Costa, P.; Sousa Lobo, J. M. Modeling and comparison of dissolution profiles. *Eur. J. Pharm. Sci.* **2001**, *13*, 123–133, doi:http://dx.doi.org/10.1016/S0928-0987(01)00095-1.
6. Yarce, C. J.; Pineda, D.; Correa, C. E.; Salamanca, C. H. Relationship between surface properties and in Vitro drug release from a compressed matrix containing an amphiphilic polymer material. *Pharmaceuticals* **2016**, *9*, doi:10.3390/ph9030034.
7. Hall, E. Fick ′ s Diffusion Experiments Revisited. *Med. Phys.* **2014**, 1–44,

doi:10.4236/ahs.2014.34017.
8. Ritger, P. L.; Peppas, N. A. A simple equation for description of solute release II. Fickian and anomalous release from swellable devices. *J. Control. Release* **1987**, *5*, 37–42, doi:10.1016/0168-3659(87)90035-6.
9. Salamanca, C. H.; Yarce, C. J.; Zapata, C. A.; Giraldo, J. A. Relationship between the polymeric ionization degree and powder and surface properties in materials derived from poly(maleic anhydride-alt-octadecene). *Molecules* **2018**, *23*, doi:10.3390/molecules23020320.
10. Talukdar, M. M.; Kinget, R. Swelling and Drug-Release Behavior of Xanthan Gum Matrix Tablets. *Int. J. Pharm.* **1995**, *120*, 63–72.
11. Chang, C.; He, M.; Zhou, J.; Zhang, L. Swelling behaviors of pH- and salt-responsive cellulose-based hydrogels. *Macromolecules* **2011**, *44*, 1642–1648, doi:10.1021/ma102801f.
12. Serra, L.; Doménech, J.; Peppas, N. A. Drug transport mechanisms and release kinetics from molecularly designed poly(acrylic acid-g-ethylene glycol) hydrogels. *Biomaterials* **2006**, *27*, 5440–5451, doi:10.1016/j.biomaterials.2006.06.011.
13. Berg, M. C.; Zhai, L.; Cohen, R. E.; Rubner, M. F. Controlled drug release from porous polyelectrolyte multilayers. *Biomacromolecules* **2006**, *7*, 357–64, doi:10.1021/bm050174e.
14. Sujja-Areevath, J.; Munday, D. L.; Cox, P. J.; Khan, K. A. Relationship between swelling, erosion and drug release in hydrophillic natural gum mini-matrix formulations. *Eur. J. Pharm. Sci.* **1998**, *6*, 207–217, doi:10.1016/S0928-0987(97)00072-9.
15. 5 - Mathematical models of drug release. In *Strategies to Modify the Drug Release from Pharmaceutical Systems*; Bruschi, M. L., Ed.; Woodhead Publishing, 2015; pp. 63–86 ISBN 978-0-08-100092-2.
16. Katzhendler, I.; Hoffman, A.; Goldberger, A.; Friedman, M. Modeling of Drug Release from Erodible Tablets. *J. Pharm. Sci.* **1997**, *86*, 110–115, doi:10.1021/js9600538.
17. Hopfenberg, H. B.; Hsu, K. C. Swelling-controlled, constant rate delivery systems. *Polym. Eng. Sci.* **1978**, *18*, 1186–1191, doi:10.1002/pen.760181511.
18. Costa, P.; Sousa Lobo, J. M. Modeling and comparison of dissolution profiles. *Eur. J. Pharm. Sci.* 2001, *13*, 123–133.
19. Jalal, I.; Zmaily, E.; Najib, N. Dissolution kinetics of commercially available controlled-release theophylline preparations. *Int. J. Pharm.* **1989**, *52*, 63–70, doi:10.1016/0378-5173(89)90089-6.
20. Singh, B.; Sharma, V. Influence of polymer network parameters of tragacanth gum-based pH responsive hydrogels on drug delivery. *Carbohydr. Polym.* **2014**, *101*, 928–940, doi:10.1016/j.carbpol.2013.10.022.
21. Özkan, Y.; Özalp, Y.; Savaşer, A.; Özkan, S. A. Comparative dissolution testing of paracetamol commercial tablet dosage forms. *Acta Pol. Pharm. - Drug Res.* **2000**, *57*, 33–41.
22. Salome, A. C.; Godswill, C. O.; Ikechukwu, I. O. Kinetics and mechanisms of drug release from swellable and non swellable matrices: A review. *Res. J. Pharm. Biol. Chem. Sci.* **2013**, *4*, 97–103, doi:10.3390/polym6092451.
